# The genome sequence of the European harvest mouse, *Micromys minutus *(Pallas, 1771)

**DOI:** 10.12688/wellcomeopenres.22747.1

**Published:** 2024-07-22

**Authors:** Michelle F. O'Brien, Rosa Lopez Colom, Natural History Museum Genome Acquisition Lab, Darwin Tree of Life Barcoding collective, Wellcome Sanger Institute Tree of Life Management, Samples and Laboratory team, Wellcome Sanger Institute Scientific Operations: Sequencing Operations, Wellcome Sanger Institute Tree of Life Core Informatics team, Tree of Life Core Informatics collective, Darwin Tree of Life Consortium

**Affiliations:** 1 Wildfowl and Wetlands Trust, Slimbridge, England, UK

**Keywords:** Micromys minutus, European harvest mouse, genome sequence, chromosomal, Rodentia

## Abstract

We present a genome assembly from an individual female *Micromys minutus* (the European harvest mouse; Chordata; Mammalia; Rodentia; Muridae). The genome sequence spans 2,651.80 megabases. Most of the assembly is scaffolded into 34 chromosomal pseudomolecules, including the X sex chromosome. The mitochondrial genome has also been assembled and is 16.24 kilobases in length.

## Species taxonomy

Eukaryota; Opisthokonta; Metazoa; Eumetazoa; Bilateria; Deuterostomia; Chordata; Craniata; Vertebrata; Gnathostomata; Teleostomi; Euteleostomi; Sarcopterygii; Dipnotetrapodomorpha; Tetrapoda; Amniota; Mammalia; Theria; Eutheria; Boreoeutheria; Euarchontoglires; Glires; Rodentia; Myomorpha; Muroidea; Muridae; Murinae; *Micromys*; *Micromys minutus* (Pallas, 1771) (NCBI:txid13151).

## Background

The European Harvest mouse ( *Micromys minutus*) is a very small mouse (5–8 cm in length and weighing 5–8 g), with orange to brown fur dorsally and white fur on the underside. It has a round face with a blunt nose ( Burnie, 2001). Females give birth to 2 to 6 young after a 21-day pregnancy ( Burnie, 2001). Young are weaned around day 18 and sexual maturity is achieved at 6 to 7 weeks old ( Wilson *et al.*, 2017). This is the only “old-world” rodent with a prehensile tail, and it is omnivorous – feeding mostly on seeds, fruits and occasionally fungi, moss and small invertebrates. This species constructs spherical nests in long grasses or similar vegetation ( Burnie, 2001).

It lives at elevations up to 1 700 m and most often lives among reeds, building nests 20–50 cm above the ground. It can also be found in cereal fields during the summer months and after harvest, will retreat to live in hedgerows alongside fields ( Wilson *et al.,* 2017). European harvest mice are usually nocturnal but can sometimes be active during the day as well. This species is terrestrial and very good at climbing. In winter it can produce burrows in the soil ( Wilson *et al*., 2017). These burrows can affect soil structure and moisture content leading to impacts on plants and invertebrates.

The species distribution is from Great Britain and northern Spain across Europe, Russia and Asia to Laos and Vietnam ( Wilson *et al.*, 2017). Although *Micromys minutus* is listed as Least Concern globally in the IUCN Red list, within the UK it is listed as Near Threatened in Great Britain, Least Concern in England, Critically Endangered in Scotland and Vulnerable in Wales ( Mathews & Harrower, 2020).

Harvest mice are thought to be indicators of ecosystem health and play an important role in their habitats ( Occhiuto *et al.*, 2021). They also have an important place in food chains as they are preyed upon by numerous predators such as birds of prey, foxes, snakes etc. and play an important role in the diets of these species ( Darinot, 2016).

Previous studies have investigated the spatial scale and the landscape determinants of genetic structure in this wetland species ( Darinot, 2016). At a regional scale a strong genetic differentiation was shown between populations – and allelic richness and heterozygosity indices, reflecting the genetic diversity of populations, were the highest in populations located in very large wetlands associated with numerous suitable habitats.

The genome of the European harvest mouse, *Micromys minutus*, was sequenced as part of the Darwin Tree of Life Project, a collaborative effort to sequence all named eukaryotic species in the Atlantic Archipelago of Britain and Ireland.

## Genome sequence report

**Figure 1.**
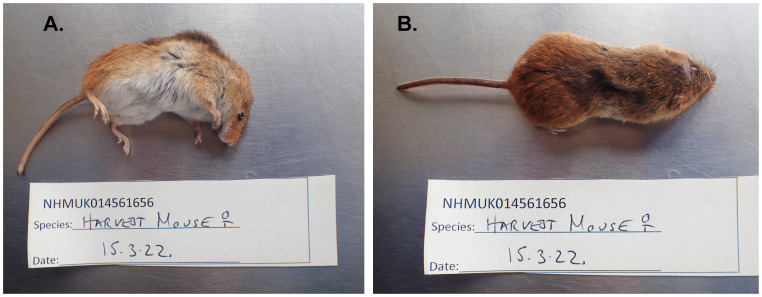
Photographs of the *Micromys minutus* (mMicMin1) specimen used for genome sequencing.

The genome of an adult female *Micromys minutus* ( Figure 1) was sequenced using Pacific Biosciences single-molecule HiFi long reads, generating a total of 29.07 Gb (gigabases) from 2.47 million reads, providing approximately 28-fold coverage. Primary assembly contigs were scaffolded with chromosome conformation Hi-C data, which produced 418.30 Gbp from 2,770.20 million reads, yielding an approximate coverage of 158-fold. Specimen and sequencing information is summarised in Table 1.

**Table 1.** Specimen and sequencing data for *Micromys minutus*.

Project information			
**Study title**	Micromys minutus (European harvest mouse)		
**Umbrella BioProject**	PRJEB71536		
**Species**	*Micromys minutus*		
**BioSample**	SAMEA112468042		
**NCBI taxonomy ID**	13151		
Specimen information			
Technology	ToLID	BioSample accession	Organism part
**PacBio long read sequencing**	mMicMin1	SAMEA112468110	muscle
**Hi-C sequencing**	mMicMin1	SAMEA112468111	muscle
**RNA sequencing**	mMicMin1	SAMEA112468110	muscle
Sequencing information			
Platform	Run accession	Read count	Base count (Gb)
**Hi-C Illumina NovaSeq 6000**	ERR12512731	2.77e+09	418.3
**PacBio Revio**	ERR12408786	6.08e+06	59.35
**PacBio Sequel IIe**	ERR12408787	2.47e+06	29.07
**RNA Illumina NovaSeq 6000**	ERR12512732	5.67e+07	8.55

**Figure 2.**
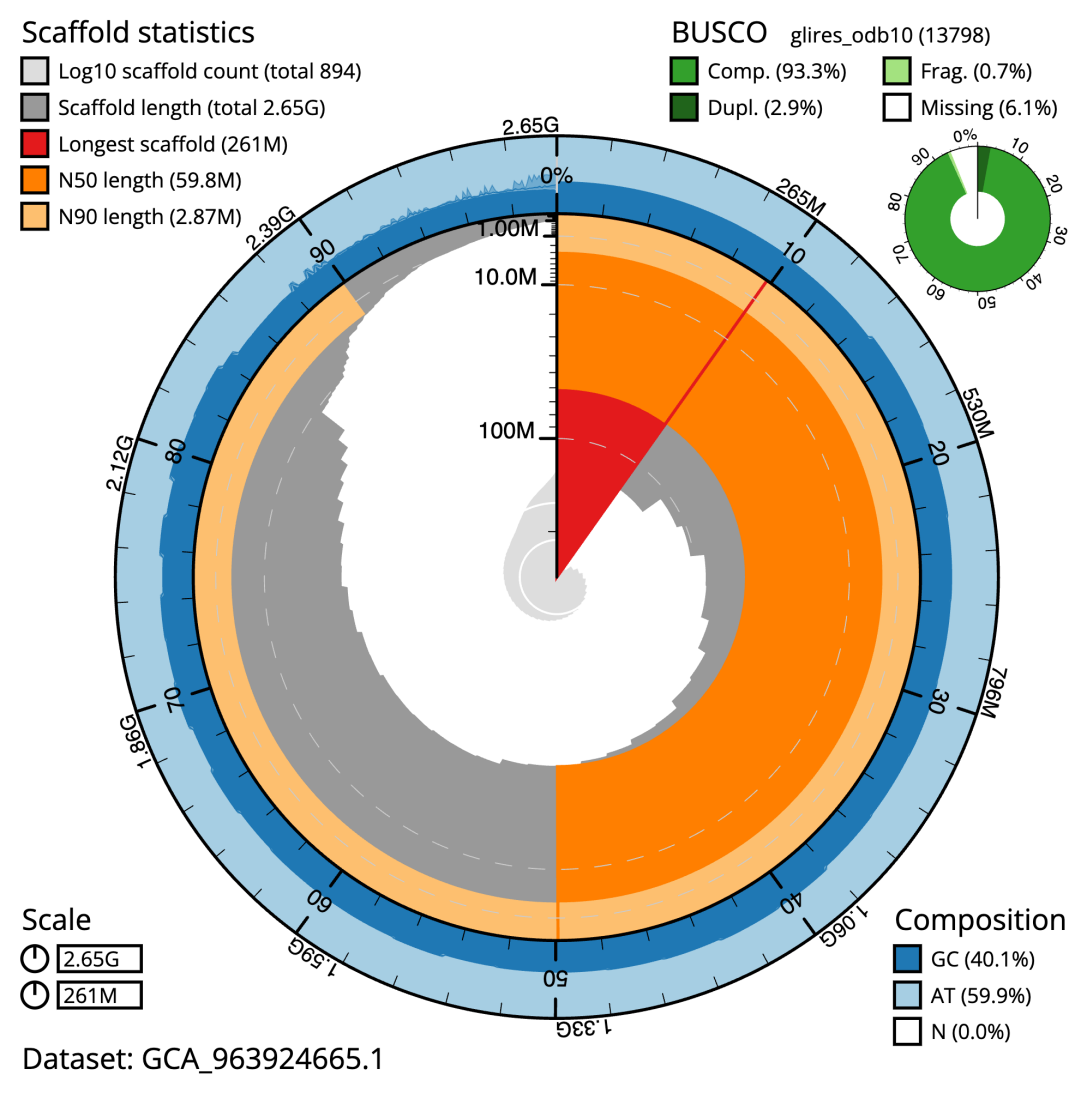
Genome assembly of *Micromys minutus*, mMicMin1.1: metrics. The BlobToolKit snail plot shows N50 metrics and BUSCO gene completeness. The main plot is divided into 1,000 size-ordered bins around the circumference with each bin representing 0.1% of the 2,651,787,409 bp assembly. The distribution of scaffold lengths is shown in dark grey with the plot radius scaled to the longest scaffold present in the assembly (261,318,752 bp, shown in red). Orange and pale-orange arcs show the N50 and N90 scaffold lengths (59,776,753 and 2,866,000 bp), respectively. The pale grey spiral shows the cumulative scaffold count on a log scale with white scale lines showing successive orders of magnitude. The blue and pale-blue area around the outside of the plot shows the distribution of GC, AT and N percentages in the same bins as the inner plot. A summary of complete, fragmented, duplicated and missing BUSCO genes in the glires_odb10 set is shown in the top right. An interactive version of this figure is available at https://blobtoolkit.genomehubs.org/view/Micromys_minutus/dataset/GCA_963924665.1/snail.

**Figure 3.**
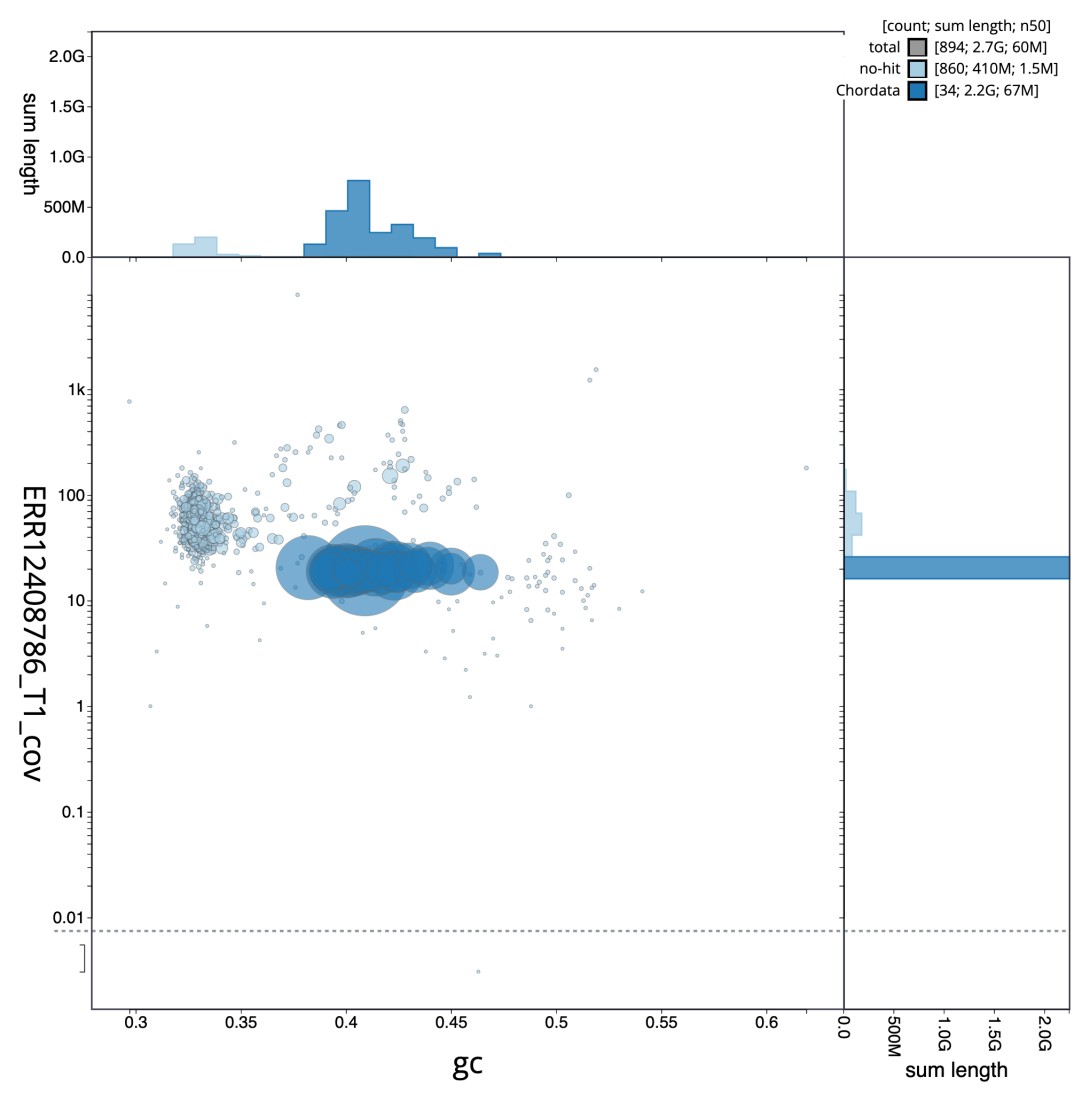
Genome assembly of *Micromys minutus*, mMicMin1.1: BlobToolKit GC-coverage plot. Sequences are coloured by phylum. Circles are sized in proportion to sequence length. Histograms show the distribution of sequence length sum along each axis. An interactive version of this figure is available at https://blobtoolkit.genomehubs.org/view/Micromys_minutus/dataset/GCA_963924665.1/blob.

**Figure 4.**
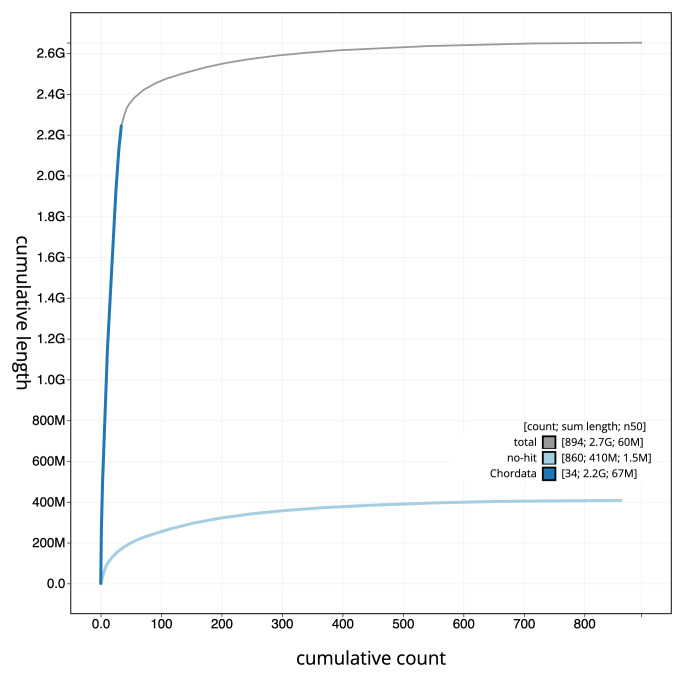
Genome assembly of *Micromys minutus* mMicMin1.1: BlobToolKit cumulative sequence plot. The grey line shows cumulative length for all sequences. Coloured lines show cumulative lengths of sequences assigned to each phylum using the buscogenes taxrule. An interactive version of this figure is available at https://blobtoolkit.genomehubs.org/view/Micromys_minutus/dataset/GCA_963924665.1/cumulative.

**Figure 5.**
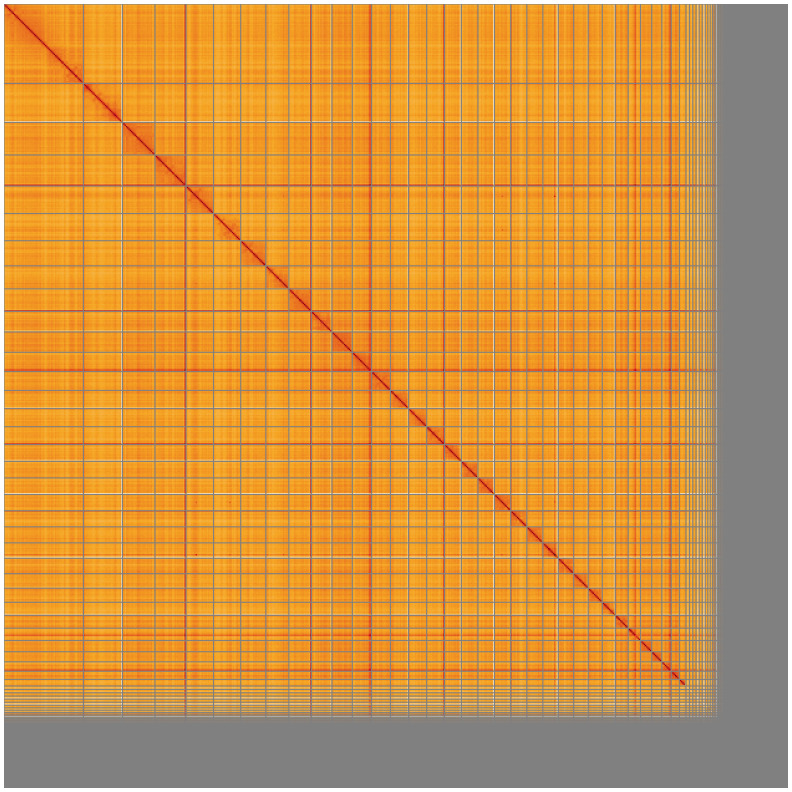
Genome assembly of *Micromys minutus* mMicMin1.1: Hi-C contact map of the mMicMin1.1 assembly, visualised using HiGlass. Chromosomes are shown in order of size from left to right and top to bottom. An interactive version of this figure may be viewed at https://genome-note-higlass.tol.sanger.ac.uk/l/?d=ASZwPyTqTdiSPDjGvswDkw.

Manual assembly curation corrected 40 missing joins or mis-joins, reducing the scaffold number by 1.43%, and increasing the scaffold N50 by 1.52%. The final assembly has a total length of 2,651.80 Mb in 893 sequence scaffolds with a scaffold N50 of 59.8 Mb ( Table 2). The total count of gaps in the scaffolds is 2,020, with an average length of 200.00 bp. The snail plot in Figure 2 provides a summary of the assembly statistics, while the distribution of assembly scaffolds on GC proportion and coverage is shown in Figure 3. The cumulative assembly plot in Figure 4 shows curves for subsets of scaffolds assigned to different phyla. Most (84.97%) of the assembly sequence was assigned to 34 chromosomal-level scaffolds, representing 33 autosomes and the X sex chromosome. Chromosome-scale scaffolds confirmed by the Hi-C data are named in order of size ( Figure 5; Table 3). The X chromosome was identified based on synteny to *Mus musculus*. While not fully phased, the assembly deposited is of one haplotype. Contigs corresponding to the second haplotype have also been deposited. The mitochondrial genome was also assembled and can be found as a contig within the multifasta file of the genome submission.

**Table 2.** Genome assembly data for *Micromys minutus*, mMicMin1.1.

Genome assembly		
Assembly name	mMicMin1.1	
Assembly accession	GCA_963924665.1	
*Accession of alternate haplotype*	*GCA_963924645.1*	
Span (Mb)	2,651.80	
Number of contigs	2,914	
Contig N50 length (Mb)	1.8	
Number of scaffolds	893	
Scaffold N50 length (Mb)	59.8	
Longest scaffold (Mb)	261.32	
Assembly metrics *		*Benchmark*
Consensus quality (QV)	62.2	*≥ 50*
*k*-mer completeness	100.0%	*≥ 95%*
BUSCO **	C:93.2%[S:90.3%,D:2.9%], F:0.8%,M:6.0%,n:13,798	*C ≥ 95%*
Percentage of assembly mapped to chromosomes	84.97%	*≥ 95%*
Sex chromosomes	XX	*localised homologous pairs*
Organelles	Mitochondrial genome: 16.24 kb	*complete single alleles*

* Assembly metric benchmarks are adapted from column VGP-2020 of “Table 1: Proposed standards and metrics for defining genome assembly quality” from Rhie *et al.* (2021).

** BUSCO scores based on the glires_odb10 BUSCO set using version 5.4.3. C = complete [S = single copy, D = duplicated], F = fragmented, M = missing, n = number of orthologues in comparison. A full set of BUSCO scores is available at https://blobtoolkit.genomehubs.org/view/Micromys_minutus/dataset/GCA_963924665.1/busco.

**Table 3.** Chromosomal pseudomolecules in the genome assembly of *Micromys minutus*, mMicMin1.

INSDC accession	Name	Length (Mb)	GC%
OZ004780.1	1	261.32	41.0
OZ004782.1	2	107.09	42.5
OZ004783.1	3	102.31	41.5
OZ004784.1	4	90.24	40.0
OZ004785.1	5	89.61	39.5
OZ004786.1	6	82.73	40.0
OZ004787.1	7	75.8	39.5
OZ004788.1	8	73.94	40.5
OZ004789.1	9	67.81	44.0
OZ004790.1	10	66.95	45.0
OZ004791.1	11	63.58	43.0
OZ004792.1	12	61.79	42.0
OZ004793.1	13	59.78	40.5
OZ004794.1	14	59.63	40.0
OZ004795.1	15	58.88	39.5
OZ004796.1	16	55.3	42.5
OZ004797.1	17	54.52	41.0
OZ004798.1	18	54.41	41.0
OZ004799.1	19	53.42	40.5
OZ004800.1	20	53.0	39.5
OZ004801.1	21	52.78	42.5
OZ004802.1	22	47.8	41.5
OZ004803.1	23	50.83	39.5
OZ004804.1	24	50.63	43.5
OZ004805.1	25	46.29	42.5
OZ004806.1	26	42.87	39.0
OZ004807.1	27	42.37	44.0
OZ004808.1	28	40.63	40.0
OZ004809.1	29	36.83	46.5
OZ004810.1	30	33.23	42.0
OZ004811.1	31	30.99	43.5
OZ004812.1	32	26.91	45.0
OZ004813.1	33	21.6	40.0
OZ004781.1	X	128.38	38.0
OZ004814.1	MT	0.02	37.5

The estimated Quality Value (QV) of the final assembly is 62.2 with *k*-mer completeness of 100.0%, and the assembly has a BUSCO v5.4.3 completeness of 93.2% (single = 90.3%, duplicated = 2.9%), using the glires_odb10 reference set ( *n* = 13,798).

Metadata for specimens, BOLD barcode results, spectra estimates, sequencing runs, contaminants and pre-curation assembly statistics are given at https://tolqc.cog.sanger.ac.uk/darwin/mammals/Micromys_minutus/.

## Methods

### Sample acquisition

The samples used for sequencing were taken from a deceased captive adult female *Micromys minutus* (specimen ID NHMUK014561656, ToLID mMicMin1) that was presented for post-mortem examination. Muscle tissue samples were taken from areas of the thigh and the back. The specimen was collected and identified by Michelle O'Brien (Wildfowl Wetlands Trust) and preserved by dry freezing at –80 °C.

In addition to identification based on morphology, the species taxonomy was verified by DNA barcoding soon after collection, according to the framework developed by Twyford *et al*. (2024). A small sample was dissected from the specimens and stored in ethanol. The tissue was lysed, and the COI marker region was amplified by PCR. Amplicons were sequenced and compared to the BOLD database, confirming the species identification ( Crowley *et al.*, 2023). The standard operating procedures for the Darwin Tree of Life barcoding have been deposited on protocols.io ( Beasley *et al.*, 2023). The remaining parts of the specimen were shipped on dry ice to the Wellcome Sanger Institute (WSI). A DNA barcode was also generated from the PacBio sequencing data at a later stage for sample tracking through the genome production pipeline at the WSI ( Twyford *et al.*, 2024).

### Nucleic acid extraction

The workflow for high molecular weight (HMW) DNA extraction at the WSI Tree of Life Core Laboratory includes a sequence of core procedures: sample preparation; sample homogenisation, DNA extraction, fragmentation, and clean-up. In sample preparation, the mMicMin1 sample was weighed and dissected on dry ice ( Jay *et al.*, 2023). For sample homogenisation, muscle tissue was cryogenically disrupted using the Covaris cryoPREP ^®^ Automated Dry Pulverizer ( Narváez-Gómez *et al.*, 2023). HMW DNA was extracted using the Automated MagAttract v2 protocol ( Oatley *et al.*, 2023a). DNA was sheared into an average fragment size of 12–20 kb in a Megaruptor 3 system with speed setting 31 ( Bates *et al.*, 2023). Sheared DNA was purified by solid-phase reversible immobilisation ( Oatley *et al.*, 2023b): in brief, the method employs a 1.8X ratio of AMPure PB beads to sample to eliminate shorter fragments and concentrate the DNA. The concentration of the sheared and purified DNA was assessed using a Nanodrop spectrophotometer and Qubit Fluorometer using the Qubit dsDNA High Sensitivity Assay kit. Fragment size distribution was evaluated by running the sample on the FemtoPulse system.

RNA was extracted from muscle tissue of mMicMin1 in the Tree of Life Laboratory at the WSI using the RNA Extraction: Automated MagMax™ *mir*Vana protocol ( do Amaral *et al.*, 2023). The RNA concentration was assessed using a Nanodrop spectrophotometer and a Qubit Fluorometer using the Qubit RNA Broad-Range Assay kit. Analysis of the integrity of the RNA was done using the Agilent RNA 6000 Pico Kit and Eukaryotic Total RNA assay.

Protocols developed by the WSI Tree of Life laboratory are publicly available on protocols.io ( Denton *et al.*, 2023).

### Sequencing

Pacific Biosciences HiFi circular consensus DNA sequencing libraries were constructed according to the manufacturers’ instructions. Poly(A) RNA-Seq libraries were constructed using the NEB Ultra II RNA Library Prep kit. DNA and RNA sequencing was performed by the Scientific Operations core at the WSI on Pacific Biosciences Sequel IIe (HiFi) and Illumina NovaSeq 6000 (RNA-Seq) instruments. Hi-C data were also generated from muscle tissue of mMicMin1 using the Arima-HiC v2 kit. The Hi-C sequencing was performed using paired-end sequencing with a read length of 150 bp on the Illumina NovaSeq 6000 instrument.

### Genome assembly, curation and evaluation

***Assembly***

HiFi reads were assembled using the ‘sanger-tol/genomeassembly’ pipeline ( Krasheninnikova *et al.*, 2024). Original assembly of HiFi reads is performed using Hifiasm ( Cheng *et al.*, 2021) with the --primary option. Haplotypic duplications were identified and removed with purge_dups ( Guan *et al.*, 2020). Hi-C reads are further mapped with bwamem2 ( Vasimuddin *et al.*, 2019) to the primary contigs, which are further scaffolded using the provided Hi-C data ( Rao *et al.*, 2014) in YaHS ( Zhou *et al.*, 2023) using the --break option. Scaffolded assemblies are evaluated using Gfastats ( Formenti *et al.*, 2022), BUSCO ( Manni *et al.*, 2021) and MERQURY.FK ( Rhie *et al.*, 2020).

The mitochondrial genome was assembled using MitoHiFi ( Uliano-Silva *et al.*, 2023), which runs MitoFinder ( Allio *et al.*, 2020) or MITOS ( Bernt *et al.*, 2013) and uses these annotations to select the final mitochondrial contig and to ensure the general quality of the sequence.

***Assembly curation***

The assembly was decontaminated using the Assembly Screen for Cobionts and Contaminants (ASCC) pipeline (article in preparation). Flat files and maps used in curation were generated in TreeVal ( Pointon *et al.*, 2023). Manual curation was primarily conducted using PretextView ( Harry, 2022), with additional insights provided by JBrowse2 ( Diesh *et al.*, 2023) and HiGlass ( Kerpedjiev *et al.*, 2018). Scaffolds were visually inspected and corrected as described by Howe *et al*. (2021). Any identified contamination, missed joins, and mis-joins were corrected, and duplicate sequences were tagged and removed. The sex chromosome was identified by synteny with *Mus musculus*. The entire process is documented at https://gitlab.com/wtsi-grit/rapid-curation (article in preparation).

***Evaluation of the final assembly***

The final assembly was post-processed and evaluated with the three Nextflow ( Di Tommaso *et al.*, 2017) DSL2 pipelines “sanger-tol/readmapping” ( Surana *et al.*, 2023a), “sanger-tol/genomenote” ( Surana *et al.*, 2023b), and “sanger-tol/blobtoolkit” ( Muffato *et al.*, 2024). The pipeline sanger-tol/readmapping aligns the Hi-C reads with bwa-mem2 ( Vasimuddin *et al.*, 2019) and combines the alignment files with SAMtools ( Danecek *et al.*, 2021). The sanger-tol/genomenote pipeline transforms the Hi-C alignments into a contact map with BEDTools ( Quinlan & Hall, 2010) and the Cooler tool suite ( Abdennur & Mirny, 2020), which is then visualised with HiGlass ( Kerpedjiev *et al.*, 2018). It also provides statistics about the assembly with the NCBI datasets ( Sayers *et al.*, 2024) report, computes *k*-mer completeness and QV consensus quality values with FastK and MERQURY.FK, and a completeness assessment with BUSCO ( Manni *et al.*, 2021).

The sanger-tol/blobtoolkit pipeline is a Nextflow port of the previous Snakemake Blobtoolkit pipeline ( Challis *et al.*, 2020). It aligns the PacBio reads with SAMtools and minimap2 ( Li, 2018) and generates coverage tracks for regions of fixed size. In parallel, it queries the GoaT database ( Challis *et al.*, 2023) to identify all matching BUSCO lineages to run BUSCO ( Manni *et al.*, 2021). For the three domain-level BUSCO lineage, the pipeline aligns the BUSCO genes to the Uniprot Reference Proteomes database ( Bateman *et al.*, 2023) with DIAMOND ( Buchfink *et al.*, 2021) blastp. The genome is also split into chunks according to the density of the BUSCO genes from the closest taxonomically lineage, and each chunk is aligned to the Uniprot Reference Proteomes database with DIAMOND blastx. Genome sequences that have no hit are then chunked with seqtk and aligned to the NT database with blastn ( Altschul *et al.*, 1990). All those outputs are combined with the blobtools suite into a blobdir for visualisation.

The genome assembly and evaluation pipelines were developed using the nf-core tooling ( Ewels *et al.*, 2020), use MultiQC ( Ewels *et al.*, 2016), and make extensive use of the Conda package manager, the Bioconda initiative ( Grüning *et al.*, 2018), the Biocontainers infrastructure ( da Veiga Leprevost *et al.*, 2017), and the Docker ( Merkel, 2014) and Singularity ( Kurtzer *et al.*, 2017) containerisation solutions. Table 4 contains a list of relevant software tool versions and sources.

**Table 4.** Software tools: versions and sources.

Software tool	Version	Source
BEDTools	2.30.0	https://github.com/arq5x/bedtools2
BLAST	2.14.0	ftp://ftp.ncbi.nlm.nih.gov/blast/executables/blast+/
BlobToolKit	4.3.7	https://github.com/blobtoolkit/blobtoolkit
BUSCO	5.4.3 and 5.5.0	https://gitlab.com/ezlab/busco
bwa-mem2	2.2.1	https://github.com/bwa-mem2/bwa-mem2
Cooler	0.8.11	https://github.com/open2c/cooler
DIAMOND	2.1.8	https://github.com/bbuchfink/diamond
fasta_windows	0.2.4	https://github.com/tolkit/fasta_windows
FastK	427104ea91c78c3b8b8b49f1a7d6bbeaa869ba1c	https://github.com/thegenemyers/FASTK
Gfastats	1.3.6	https://github.com/vgl-hub/gfastats
GoaT CLI	0.2.5	https://github.com/genomehubs/goat-cli
Hifiasm	0.19.8-r603	https://github.com/chhylp123/hifiasm
HiGlass	44086069ee7d4d3f6f3f0012569789ec138f42b84aa44357826c0b6753eb28de	https://github.com/higlass/higlass
Merqury.FK	d00d98157618f4e8d1a9190026b19b471055b22e	https://github.com/thegenemyers/MERQURY.FK
MitoHiFi	3	https://github.com/marcelauliano/MitoHiFi
MultiQC	1.14, 1.17, and 1.18	https://github.com/MultiQC/MultiQC
NCBI Datasets	15.12.0	https://github.com/ncbi/datasets
Nextflow	23.04.0-5857	https://github.com/nextflow-io/nextflow
PretextView	0.2	https://github.com/sanger-tol/PretextView
purge_dups	1.2.5	https://github.com/dfguan/purge_dups
samtools	1.16.1, 1.17, and 1.18	https://github.com/samtools/samtools
sanger-tol/ascc	-	https://github.com/sanger-tol/ascc
sanger-tol/genomeassembly	0.10.0	https://github.com/sanger-tol/genomeassembly
sanger-tol/genomenote	1.1.1	https://github.com/sanger-tol/genomenote
sanger-tol/readmapping	1.2.1	https://github.com/sanger-tol/readmapping
Seqtk	1.3	https://github.com/lh3/seqtk
Singularity	3.9.0	https://github.com/sylabs/singularity
TreeVal	1.0.0	https://github.com/sanger-tol/treeval
YaHS	1.2a.2	https://github.com/c-zhou/yahs

Table 4 contains a list of relevant software tool versions and sources.

### Wellcome Sanger Institute – Legal and Governance

The materials that have contributed to this genome note have been supplied by a Darwin Tree of Life Partner. The submission of materials by a Darwin Tree of Life Partner is subject to the **‘Darwin Tree of Life Project Sampling Code of Practice’**, which can be found in full on the Darwin Tree of Life website here. By agreeing with and signing up to the Sampling Code of Practice, the Darwin Tree of Life Partner agrees they will meet the legal and ethical requirements and standards set out within this document in respect of all samples acquired for, and supplied to, the Darwin Tree of Life Project. 

Further, the Wellcome Sanger Institute employs a process whereby due diligence is carried out proportionate to the nature of the materials themselves, and the circumstances under which they have been/are to be collected and provided for use. The purpose of this is to address and mitigate any potential legal and/or ethical implications of receipt and use of the materials as part of the research project, and to ensure that in doing so we align with best practice wherever possible. The overarching areas of consideration are: 

●   Ethical review of provenance and sourcing of the material

●   Legality of collection, transfer and use (national and international)

Each transfer of samples is further undertaken according to a Research Collaboration Agreement or Material Transfer Agreement entered into by the Darwin Tree of Life Partner, Genome Research Limited (operating as the Wellcome Sanger Institute), and in some circumstances other Darwin Tree of Life collaborators.

## Data Availability

European Nucleotide Archive: Micromys minutus (European harvest mouse). Accession number PRJEB71536; https://identifiers.org/ena.embl/PRJEB71536 ( Wellcome Sanger Institute, 2024). The genome sequence is released openly for reuse. The *Micromys minutus* genome sequencing initiative is part of the Darwin Tree of Life (DToL) project. All raw sequence data and the assembly have been deposited in INSDC databases. The genome will be annotated using available RNA-Seq data and presented through the Ensembl pipeline at the European Bioinformatics Institute. Raw data and assembly accession identifiers are reported in Table 1 and Table 2.

## Author information

Members of the University of Oxford and Wytham Woods Genome Acquisition Lab are listed here: https://doi.org/10.5281/zenodo.12157525.

Members of the Darwin Tree of Life Barcoding collective are listed here: https://doi.org/10.5281/zenodo.12158331

Members of the Wellcome Sanger Institute Tree of Life Management, Samples and Laboratory team are listed here: https://doi.org/10.5281/zenodo.12162482.

Members of Wellcome Sanger Institute Scientific Operations: Sequencing Operations are listed here: https://doi.org/10.5281/zenodo.12165051.

Members of the Wellcome Sanger Institute Tree of Life Core Informatics team are listed here: https://doi.org/10.5281/zenodo.12160324.

Members of the Tree of Life Core Informatics collective are listed here: https://doi.org/10.5281/zenodo.12205391.

Members of the Darwin Tree of Life Consortium are listed here: https://doi.org/10.5281/zenodo.4783558.
