# Supplementary material for: The genome sequence of the European harvest mouse, Micromys minutus (Pallas, 1771)
Source: Wellcome Open Res. 2024 Jul 22;9:394. [Version 1] doi: 10.12688/wellcomeopenres.22747.1 (PMC11474158; doi:10.12688/wellcomeopenres.22747.1)
Supplement: Peer review history

# Reviewer report: Reviewer response for version 1

**DOI:** 10.21956/wellcomeopenres.25050.r102137

**Published:** 2024-10-14

**Authors:** Naoki Osada

**Affiliations:** 1 Hokkaido University, Sapporo, Japan

In this manuscript, the authors present a new genome assembly of the European harvest mouse ( *Micromys minutus*) and provide key statistics to evaluate the quality of the assembled genome sequence. The manuscript is clearly written, and the study appears to be appropriately performed using standardized methods.

**I have two major and one minor comment that should be addressed before final indexing:**

1. The geographic origin of the sequenced individual is not mentioned in the Methods section. Even if the exact location and date of sampling are unknown, this information should be provided as much as possible. Given that genetic variation within species is not negligible, such details are important for contextualizing the results.
2. The Introduction provides little information about previous genetic studies of this species. There has been research on karyotypes, and it is known that the number of chromosomes for this species is 2n=68. This information is crucial for evaluating the assembly status of the genome sequence, as it can provide a basis for assessing completeness and accuracy.
3. The term “X sex chromosome” appears a few times in the manuscript. It should be revised to simply “X chromosome.”

Are sufficient details of methods and materials provided to allow replication by others?

No

Is the rationale for creating the dataset(s) clearly described?

Yes

Are the datasets clearly presented in a useable and accessible format?

Yes

Are the protocols appropriate and is the work technically sound?

Yes

Reviewer Expertise:

Genome Science, Molecular Evolution, Population Genetics

I confirm that I have read this submission and believe that I have an appropriate level of expertise to confirm that it is of an acceptable scientific standard.

# Reviewer report: Reviewer response for version 1

**DOI:** 10.21956/wellcomeopenres.25050.r99395

**Published:** 2024-10-04

**Authors:** Jonathan J Hughes

**Affiliations:** 1 University of California, Riverside, USA

The authors have assembled a chromosome-scale assembly for the European harvest mouse, with benchmarking scores that indicate a high-quality of assembly. The report is generally clear and concise, and the methods are comprehensive and well described. Description of the dataset appears accurate.

**I have just two suggestions to improve the manuscript:**

- Paragraph 4 of the Background is not well written. Repeating "important role/place" three times feels redundant. It's not clear what the important role harvest mice play in their habitat is from the text. It would be great to see more detail as to why they are considered indicators of ecosystem health. This would also help give better justification for why this genome was sequenced beyond just being part of the DTOL project. 
- The authors should briefly cite some of the available cytogenetic literature to support their assembly of the *M. minutus* genome into 34 chromosomes.

Are sufficient details of methods and materials provided to allow replication by others?

Yes

Is the rationale for creating the dataset(s) clearly described?

Yes

Are the datasets clearly presented in a useable and accessible format?

Yes

Are the protocols appropriate and is the work technically sound?

Yes

Reviewer Expertise:

Genomics; phylogenomics; rodents

I confirm that I have read this submission and believe that I have an appropriate level of expertise to confirm that it is of an acceptable scientific standard.
